# Bee‐Derived Antioxidants as a Protective Strategy Against Doxorubicin‐Induced Ovarian Damage

**DOI:** 10.1002/cbdv.202500766

**Published:** 2025-07-22

**Authors:** Meltem Arıkan Malkoc, Serap Özer Yaman, Şafak Ersöz, Sevgi Kolaylı

**Affiliations:** ^1^ Vocational School of Health Sciences Karadeniz Technical University Trabzon Türkiye; ^2^ Department of Medical Biochemistry Faculty of Medicine, University of Health Sciences Trabzon Türkiye; ^3^ Department of Medical Pathology Faculty of Medicine, Karadeniz Technical University Trabzon Türkiye; ^4^ Department of Chemistry Faculty of Science, Karadeniz Technical University Trabzon Türkiye

**Keywords:** ameliorative, antioxidant, bee products, ER stress, doxorubicin

## Abstract

Antineoplastic agents can induce tissue damage through oxidative stress mechanisms. Doxorubicin, a widely used chemotherapeutic agent, has been shown to cause permanent damage to reproductive tissues. Antioxidant‐rich dietary interventions are considered a promising approach to mitigate oxidative stress‐related damage. This study aimed to evaluate the therapeutic potential of an antioxidant‐rich bee product mixture (ARPM) in ameliorating doxorubicin‐induced ovarian injury. The ARPM, composed of honey, pollen, propolis, and royal jelly, was administered via gavage to female Sprague‐Dawley rats (180–200 g) following chronic ovarian damage induced by doxorubicin (6 mg/kg, ip). Oxidative stress markers, including superoxide dismutase (SOD), malondialdehyde (MDA), and glutathione (GSH), as well as endoplasmic reticulum (ER) stress‐related markers such as 78‐kDa glucose‐regulated protein (GRP78), inositol‐requiring enzyme 1 (IRE1), C/EBP homologous protein (CHOP), tumor necrosis factor‐alpha (TNF‐α), and caspase‐3, were simultaneously evaluated. In addition, estradiol (E2) and progesterone levels were measured, and histopathological evaluations were conducted. The mixture, rich in bioactive compounds including chrysin, pinocembrin, caffeic acid, ferulic acid, and coumaric acid, was found to significantly improve ovarian function by reducing ER stress compared to the control group. These findings suggest that ARPM may offer protective effects against doxorubicin‐induced ovarian damage through its antioxidative and anti‐ER stress properties.

## Graphical abstract

Graphical abstract of Bee‐Derived Antioxidants as a Protective Strategy Against Doxorubicin‐Induced Ovarian Damage

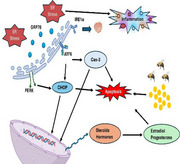

## 1 Introduction

Bee products such as honey, propolis, and bee pollen are recognized as functional foods due to their potent antioxidant, anti‐inflammatory, antimicrobial, and tissue‐regenerative properties. Owing to these bioactivities, they are increasingly utilized, either individually or in combination, as complementary therapeutic agents in various health‐related applications. Propolis is a resinous substance collected by bees and is usually extracted using ethanol, yielding an extract rich in phenolic compounds with significant bioactive potential. Bee pollen contains a wide range of vitamins, minerals, and bioactive molecules that contribute to its role in supporting overall health and improving energy metabolism, especially during therapeutic interventions. Royal jelly, the queen bee's primary nutrient, is widely used in complementary medicine for its nutrigenomic effects, especially in the context of enhancing fertility. As a natural source of carbohydrates and polyphenols, honey not only serves as an energy‐rich food, but also functions as a sustainable green solvent in a variety of applications [1, 2]. Although various methods have been used to induce oxidative stress in animal models, in recent years, oxidative stress induction approaches, especially through some agents used in cancer treatment, have become one of the most widely applied methods [3].

Doxorubicin (DOX) is a wide‐spectrum antitumor anthracycline to treat various cancers including, breast, lymphoma, testicular, and multiple myeloma and lung cancers [4]. The agent is known for its primary mechanisms of action in cancer treatment. First, it intercalates into the double‐stranded DNA, binding to DNA and inhibiting DNA and RNA synthesis. In addition, during DNA replication, it inhibits the topoisomerase II enzyme, causing unwinding of DNA supercoils and generating transient breaks in the DNA double helix. Failure to repair these breaks leads to cell death. DOX also increases the production of reactive oxygen species (ROS) in cells, inducing oxidative stress and damaging cellular macromolecules such as DNA, proteins, and lipids, thereby triggering cell death. Furthermore, it initiates apoptosis (programmed cell death) by activating intracellular signaling pathways and facilitating the activation of proteins involved in cell death, such as caspases. Finally, DOX can directly damage cell membranes and intracellular organelles like mitochondria, contributing further to cell death [5]. Among these mechanisms, oxidative stress is widely discussed as a major contributor to DOX‐induced toxicity. In particular, germ tissues (ovaries and testicles), which exhibit intense spermatogenesis and ovulation function, stand out as one of the tissues most adversely affected by DOX. Increased oxidative stress can lead to germ cell apoptosis, potentially resulting in infertility [6, 7]. While these effects cause apoptosis of cancer cells in primary cancer tissue, they cause various side effects in healthy tissues, kidneys, liver, cardiovascular, nerves, ovarian and testis, posing a significant clinical challenge [8]. Damage to the reproductive organs (ovaries and testicles) can cause serious problems for patients who want to have children. New solutions are needed to protect these peripheral tissues, especially in patients of childbearing age [9].

The endoplasmic reticulum (ER) is a critical organelle involved in the folding, processing, and targeting of proteins within a cell. It is highly susceptible to oxidative stress, which can disrupt its function and lead to the accumulation of misfolded proteins. Cellular stress conditions, such as oxidative stress, impair the ER's ability to maintain proteostasis, ultimately affecting overall cellular function and health [10, 11, 12]. Endoplasmic reticulum stress (ERS) is a condition caused by protein misfolding in the ovary and can adversely affect follicle and oocyte health. This contributes to the pathogenesis of various ovarian diseases such as polycystic ovary syndrome. Inhibition of ERS is a promising target for new therapeutic modalities [10]. ERS activates a signaling network known as the unfolded protein response (UPR). Excessive and prolonged ERS can lead to cellular dysfunction and death [7]. The 78‐kDa glucose regulatory protein (GRP78), inositol‐requiring enzyme 1 (IRE1), and C/EBP homologous protein (CHOP) collectively serve as pivotal regulators of ERS response. During cellular stress conditions, their principal function involves facilitating the precise folding of misfolded or aggregated proteins, thereby restoring cellular homeostasis [7, 13, 14].

There is extensive evidence in the literature demonstrating the protective/therapeutic effects of natural product‐derived molecules against DOX‐induced reproductive system toxicity [13]. Some polyphenols with strong antioxidant capacity, such as gallic acid [16], myricetin [15], hesperidin [16], and chlorogenic acid [19], have been reported to be effective in reducing this damage. Some of the studies with various natural extracts, including bee products, play an effective role in eliminating cellular oxidative damage caused by many oxidative stress sources [16, 18, 19, 20, 21].

Bee products are believed to enhance the immune system and improve reproductive health by positively influencing hormonal balance, which may contribute to better quality of egg and sperm production. The objective of this study was to investigate the effects of a formulated mixture of honey, propolis, pollen, and royal jelly on ovarian function, particularly on the ovulation capacity. This study aimed to investigate the protective effects of the formulated mixture against ovarian damage induced by ERS resulting from the administration of the chemotherapeutic agent DOX.

## 2 Materials and Methods

### 2.1 Samples and Preparations

The honey, pollen, propolis, and royal jelly used in the study were produced in cooperation with very experienced beekeepers in Türkiye. The oak (*Quercus* spp.) honey is a kind of honeydew honey, was produced in Kırklareli region in September 2023. The chestnut (*Chestnut sativa* L.) bee pollen was produced in Çaykara, Trabzon, in June 2023. The raw propolis was supplied by the Ilgaz mountain of Kastamonu, which is rich in chestnut trees. A fresh royal jelly sample was used from the trademark of Harşena (Amasya, Türkiye), a brand product produced.

A homogeneous mixture, referred to as antioxidant‐rich bee product mixture (ARPM) (propolis:honey:pollen:royal jelly), was prepared using honey, propolis, bee pollen, and royal jelly (1:10:2:1 w/w). The proportions of honey, propolis, bee pollen, and royal jelly used in this study (1:10:2:1, w/w) were not derived from literature data, but instead were optimized to obtain a palatable and consumable mixture. The highest proportion of honey (10 parts) was used not only for its high biological activity but also for its role as a green solvent, providing an effective medium for dissolving propolis, bee pollen, and royal jelly. First, an ethanolic propolis extract was prepared. A total of 250 g of frozen powdered propolis was extracted with 500 mL of 70% ethanol. The extraction process involved 3 h in an ultrasonic bath followed by 24 h of shaking at room temperature. After filtration, the solvent was evaporated using a rotary evaporator. From the resulting dark‐colored concentrated propolis extract, 10 g was taken and dissolved in 100 g of the oak honey. To this homogenized mixture, 20 g of powdered chestnut pollen and 10 g of the royal jelly were added, and the mixture was further homogenized using a mixer. The mixture was kept at +4°C.

### 2.2 Analysis of the ARPM

#### 2.2.1 Total Phenolic Content

Total phenolic content (TPC) was measured spectrophotometrically according to the Folin–Ciocalteu method [22]. For this process, 1 g of the mixture was mixed with 50 mL of 70% ethanol, filtered and analyzed. For the analysis, 25 µL of the extract and 400 mL of 0.2 N Folin–Ciocalteu reagent were mixed, diluted with 680 mL distilled water and incubated at room temperature for 3 min. After incubation, 400 mL of 10% Na_2_CO_3_ was added and kept at 25°C for 2 h. The absorbance was read at 760 nm on a spectrophotometer (Thermo Scientific Evolution TM 201, UV‐VIS Spectrophotometer, Madison, USA). The calibration curve was calculated for six different gallic acid standards prepared from 0.031 to 0.50 mg quercetin equivalent (QUE)/mL. The results were calculated as mg gallic acid equivalents using the standard curve.

#### 2.2.2 Total Flavonoid Content

Total flavonoid content (TFC) was determined according to the modified method [25]. In this modification, AlNO_3_ was used instead of AlCI_3_ since its solubility in water is very low. First, 25 µL of ethanolic extract was added to a mixture of 50 µL of 10% Al(NO_3_)_3_ and 50 µL of 1.0 M NH_4_CH_3_COO. This mixture was diluted to 3.0 mL with ethanol (99%) and incubated at 25°C for 45 min, then the absorbance was read at 415 nm. The calibration curve was calculated for six different quercetin standards prepared from 0.031 to 0.50 mg QUE/mL. The result was expressed as mg QUE/100 g extract using the standard curve.

### 2.3 Ferric Reducing Antioxidant Power Assay

The total antioxidant activity of the mixture of bee products was measured using the ferric reducing antioxidant power (FRAP) assay [26]. Freshly prepared FRAP reagent of ferric tripyridyltriazine (Fe^3+^–TPTZ), FeCl_3_, acetate buffer in 40 mM HCl, and 2.5 mL of 20 mM FeCl_3_·6H_2_O solution were mixed in a test tube. Then, 3 mL of the FRAP reagent was mixed with 100 µL of the extract for 4 min at 37°C, and the absorbance was read at 595 nm. The standard calibration curve was obtained using different concentrations of FeSO_4_·7H_2_O (from 1000 to 31.25 µmol/mL). The results were expressed as µmol FeSO_4_·7H_2_O equivalents/100 g sample.

### 2.4 DPPH Radical Scavenging Activity

The DPPH radical scavenging assay was measured by the spectrophotometric assay [27]. For this assay, first, 1.0 mL of 0.04 mg/mL DPPH radical solution and 1.0 mL of the extract were mixed. The mixture was kept in the dark for 45 min at 25°C, and the absorbance was read at 517 nm. To calculate the SC_50_ value, samples were prepared by making six different dilutions of the minimum ethanolic extract and treated with DPPH solution and then absorbed at 517 nm. The amount of extract scavenging 50% of this radical was determined from the graph as SC_50_.

### 2.5 Determination of the Phenolic Composition

The phenolic composition of the mixture was determined by HPLC equipped with photodiode array detectors (PDA) (Shimadzu Liquid Corporation LC 20AT) and a C18 column (250 mm × 4.6 mm, 5 µm; GL Sciences). The standard calibration graph was obtained using 25 phenolic standards. The mobile phase consisted of (A) 2% acetic acid in water and (B) acetonitrile: water (70:30). The volumes of the samples/standard injection were 20 µL, the column temperature was 30°C, and the flow rate was 1.0 mL/min [28].

### 2.6 Determination of 10‐HDA in Royal Jelly

10‐HDA, an important marker of royal jelly, was determined by HPLC‐UV. A stock solution of 200 µg/mL was obtained with 20 mg of pure 10‐HDA solvent (methanol/water, 1:1). Then, 0.1 g of methyl 4‐hydroxybenzoate (MHB) was dissolved in the same solvent as the internal standard. Equal volumes of MHB and 10‐HDA solutions were mixed, and a standard curve was created with the help of diluted solutions prepared at different concentrations [29]. Approximately 25 mg of lyophilized royal jelly sample was dissolved in 25 mL of solvent (methanol and ultrapure water, 50:50, v/v) and stirred for at least 20 min. The sample was then filtered with a 0.45‐µm filter and an equal volume of MHB was injected into the filtrate. The injection volume for each standard solution and sample was determined as 20 µL and the flow time as 15 min.

### 2.7 Experimental Animals

The experimental procedures were approved by the Animal Care and Ethical Committee of Karadeniz Technical University (Protocol no. 2023/12) and were carried out in accordance with the guidelines set by the US National Institutes of Health. Forty female Sprague‐Dawley rats (180 ± 20 g) were supplied from the Surgical Practice and Research Center of Karadeniz Technical University (Trabzon, Türkiye). The rats were kept in plastic cages within a well‐ventilated animal facility, maintained under a regulated light cycle of 12 h light and 12 h dark. They had unrestricted access to water and food.

Experimental animals were divided into four groups as follows. The mixture will be given 1 h after DOX administration. DOX was dissolved in serum physiological solution (0.9% NaCl). After the 21‐day administration stages were completed, animals were sacrificed by decapitation method on the 21st day.

Group 1: Control group (*n* = 7) (21 days 0.9% NaCl oral)

Group 2: DOX (*n* = 11) (6 mg/kg, ip, only 4, 11, and 18 days oral gavage)

Group 3: DOX + the mixture (*n* = 11) (6 mg/kg, ip + 400 mg/kg mixture)

Group 4: The mixture (*n* = 11) (400 mg/kg 21 day)

### 2.8 Biochemical Analyses

To evaluate ovarian function, serum levels of the reproductive hormones estradiol (E2) and progesterone (PGN) were measured using a commercial enzyme‐linked immunosorbent assay (ELISA) kit (BT Lab, Zhejiang, China). The hormone levels were reported in ng/L for estradiol and ng/mL for PGN.

### 2.9 In Ovarian Tissues

The ovarian specimens underwent homogenization at 9500 rpm in 1 mL of PBS utilizing a homogenizer (IKA, T25 Ultra‐Turrax, Staufen, Germany). Protein concentrations within the tissue samples were determined using a commercially available kit (Pierce BCA Protein Assay Kit; Thermo Scientific, Rockford, IL, USA) following the manufacturer's protocol, by measuring absorbance at 562 nm employing a microplate reader (VersaMax, Molecular Devices, Sunnyvale, CA, USA). All biochemical parameters in supernatants were expressed per mg protein. The results were expressed as ng/L and ng/mL, respectively.

The amount of malondialdehyde (MDA), indicative of lipid peroxidation, was assessed spectrophotometrically utilizing the thiobarbituric acid reactive substance (TBARS) method [30]. Briefly, 100 mg of tissue was homogenized with 1 mL of 1.15% KCl and 0.05% Triton X‐100 solution, then centrifuged at 3200 × *g* for 10 min. Absorbances of the resulting supernatants and standard (tetramethoxypropane) were read at 532 nm. MDA levels in the tissue were expressed as nmol/mg protein.

Tissue levels of ERS markers (GRP78, IRE1α, and CHOP) and the inflammatory marker tumor necrosis factor‐alpha (TNF‐α) were measured using commercially available ELISA kits (BT Lab, Zhejiang, China) following the manufacturer's instructions. Absorbance readings were taken at 450 nm using a microplate reader (VersaMax, Molecular Devices). GRP78 and IRE1α levels were expressed in pg/mg of protein, while TNF‐α and CHOP were reported in ng/mg of protein.

### 2.10 Histopathological Studies

The ovaries obtained from the study groups were fixed in 10% formaldehyde for 48 h. Following standard tissue preparation procedures, the samples were embedded in paraffin blocks. Sections 5 µm in thickness were cut from each paraffin block using a fully automated microtome (Leica RM 2255, Leica Instruments, Germany). These sections were then stained with hematoxylin and eosin (H&E). Follicle activity was assessed by counting the numbers of primary, secondary, tertiary, and atretic follicles [31]. This evaluation was conducted using an Olympus BX51 light microscope (Olympus Co., Tokyo, Japan) equipped with a digital camera attachment, Olympus DP71 (Olympus Co.). Atretic follicles were identified by the presence of autophagic vesicles and blebs in the cytoplasm of a quarter of granulosa cells, along with a discontinuous zona pellucida separating granulosa cells from adjacent cells [32, 33]. In addition, follicular cell degeneration, leukocyte, edema, and vascular congestion were evaluated in the preparations. The scoring was performed semi‐quantitatively on a scale of 0–3 (0: *no findings*; 1: *findings in less than 25% of the tissues*; 2: *findings in 25%–75% of the tissues*; 3: *findings in more than 75% of the tissues*).

### 2.11 Statistical Analysis

The statistical analyses were conducted using SPSS Statistics for Windows version 23 (IBM Corp., Armonk, NY, USA). The normal distribution of the data was assessed using the Kolmogorov–Smirnov test. All findings were calculated as mean ± standard deviation (SD). ANOVA, followed by the Tukey test for multiple comparisons, was employed to analyze the biochemical data. Histological outcomes were expressed as median and interquartile range (IQR) and analyzed utilizing the Kruskal–Wallis and Mann–Whitney *U* tests. A significance level of *p* < 0.01 was applied to determine statistical significance.

## 3 Results and Discussion

### 3.1 Bioactive Component Analyses

Some bioactive properties of the ARPM were summarized in Table 1. TPC and TFC of the mixture were determined as 5.47 mg GAE/mL and 0.48 mg QUE/mL, respectively. Due to our experience from previous bee product studies, the honey, pollen, propolis, and royal jelly used in the study were deliberately selected. Much of the mixture consists of oak (*Quercus* spp.) honey, which was used as a green solvent for the propolis extract, bee pollen, and royal jelly. The dark‐colored honeydew honey (*Queries* spp.) contains nearly two or three times higher antioxidant capacity compared to light‐colored blossom honeys [34, 35]. In addition, our previous study revealed also chestnut bee pollen has about four to five times higher antioxidant capacity than other pollen species and contains a high amount of proteins [36]. In this study, chestnut propolis, characterized by its high polyphenol content, similar to that of chestnut honey and pollen, was utilized as a rich source of polyphenols. One of the ingredients in the mixture is freshly produced lyophilized royal jelly. Royal jelly is a different bee product from honey, pollen, and propolis, and is rich in bioactive peptides and proteins and contains short‐chain hydroxy fatty acids such as 10‐HDA. It was added to the mixture due to its strengthening immune system and cell regenerating properties [20, 37, 38].

**TABLE 1:** Phenolic content and antioxidant properties of bee product mixture (ARPM).

	Total phenolic content (TPC), mg GAE/mL	Total flavonoid content (TFC), mg QUE/mL	Total antioxidant capacity (FRAP), mmol FeSO_4_·7H_2_O/mL	DPPH radical scavenging activity (SC_50_), µg/mL
ARPM	5.47 ± 0.13	0.48 ± 0.03	64.53 ± 0.22	30.20 ± 1.05

The composition of the phenolic components contained in the mixture was examined by HPLC–PDA (Table 2). All validation values (LOD, LOQ, recovery etc.) required for HPLC–PDA analysis according to the 25 phenolic standards defined and the corresponding chromatogram were given in detail in our previous article [26]. The compounds were divided into two classes: phenolic acids and flavonoids. According to the standards, caffeic acid, coumaric acid, and ferulic acid were identified as the predominant constituents among phenolic acids, whereas chrysin and pinocembrin emerged as the primary constituents within the flavonoid class. Galangin, rhamnetin, and CAPE were detected in lower amounts.

**TABLE 2:** Phenolic composition of the bee product (ARPM).

	Phenolic compounds	(µg/g mixture)
Phenolic acids	Gallic acid	—
	Protocatechuic acid	1.93
	Chlorogenic acid	—
	*p‐*OH benzoic acid	3.12
	Epicatechin	—
	Caffeic acid	35.16
	Syringic acid	—
	*m*‐OH benzoic acid	—
	Ellagic acid	5.60
	*p*‐Coumaric acid	22.58
	Ferulic acid	26.03
	*t*‐Cinnamic acid	8.53
Flavonoids	Rutin	—
	Quercetin	—
	Myricetin	—
	Daidzein	—
	Pinocembrin	44.58
	Luteolin	—
	Apigenin	—
	Hesperidin	—
	Rhamnetin	5.42
	Chrysin	43.05
	Galangin	—
	Galangin	14.12
	CAPE	5.60
	Resveratrol	—

*Note*: —, not detected.

The amount of 10‐HDA in the lyophilized royal jelly, as measured by HPLC‐UV, was found to be 4.20%. Considering the 1:14 ratio in the mixture, the concentration of 10‐HDA in the mixture was determined to be 0.3%. The mixture, which was given orally to experimental animals, is thought to contain significant amounts of sugar from honey and protein from bee pollen and royal jelly. However, these components were likely broken down during digestion, so their levels were not measured.

### 3.2 Antioxidant Properties

The total antioxidant capacity of the mixture was measured using the FRAP assay, and the result was 64.53 ± 0.22 mmol FeSO_4_·7H_2_O/mL. The antioxidant activity of the mixture was also measured by DPPH radical scavenging activity assay and the SC_50_ value was 30 µg/mL (Table 1). In our previous study, the DPPH radical scavenging activities of Anatolian propolis samples collected from 40 different regions were found to range between 9 and 22 µg/mL, indicating significant variability in their antioxidant capacities [37]. Moreover, in our study investigating bee pollens with different botanical origins, the SC_50_ values for DPPH radical scavenging activity were found to range between 800 and 3000 µg/mL [38]. These findings indicate that propolis exhibits approximately 50‐ or 100‐fold greater antioxidant capacity compared to pollen [38]. But, the total antioxidant capacity of the mixture was found to be lower than that of pure propolis extracts, but higher than that of the honey and the pollen samples, which was expected. To conclude, the antioxidant potential of a mixture containing honey, propolis, and pollen is largely determined by the relative amounts of each component, all of which are notable antioxidant sources [34, 39]. In a study comparable to ours, which evaluated the antioxidant capacities of honey, pollen, propolis, and royal jelly collected from the same geographical region, propolis was identified as the bee product with the highest antioxidant activity, followed by pollen, honey, and royal jelly, respectively [2]. Similarly, ethanolic Anatolian propolis from the Bitlis region has been reported to exhibit potent biological activities, including antidiabetic, antiepileptic, anticholinergic, and antiglaucoma effects [3].

### 3.3 ERS Biomarkers

The data of ERS markers measured in ovarian tissues of the experimental groups are summarized in Table 3. IRE1α, GRP78, and CHOP proteins were measured as three ERS markers. The amount of IRE1α, a transmembrane protein with kinase/nuclease activities in response to ERS [38, 39], was found to increase approximately fivefold in the DOX group compared to the control group but not in the mixture group. According to the study data, the expression of IRE1 transmembrane protein was found to be significantly reduced in the group given the mixture together with DOX (*p* < 0.01). IRE1 is a crucial component of the ERS response. Under ERS conditions, IRE1 detects cellular stress triggered by the accumulation of misfolded proteins. Subsequently, IRE1 becomes activated and exhibits endonuclease activity. This activity triggers the splicing of X‐box binding protein 1 (XBP1) mRNA by cleaving specific regions of mRNA within the cell. It shows that the mixture used does not increase the expression of IRE1 membrane protein, that is, it does not cause ERS. As a result, XBP1 is converted into its active form and regulates the transcription of a set of genes involved in the ERS response. This process is critical for restoring cellular homeostasis and preserving cell survival [13, 43].

**TABLE 3:** The ER stress parameters in the ovarian tissues of the rats.

Parameters	Control (saline) (*n* = 7)	DOX (6 mg/kg) (*n* = 11)	DOX + ARPM (400 mg/kg) (*n* = 11)	ARPM (400 mg/kg) (*n* = 11)
IRE1 (pg/mg protein)	147.20 ± 27.50^a^, ^b^, ^c^	830.40 ± 258.02^a^	402 ± 84.8^a^, ^b^	154 ± 21.3^b^, ^c^
GRP78 (pg/mg protein)	107.05 ± 9.63^a^, ^b^, ^c^	1818.30 ± 497.00^a^	641 ± 109^a^, ^b^	114 ± 14.9^b^, ^c^
CHOP (ng/mg protein)	2.09 ± 0.81^a^, ^b^, ^c^	26.20 ± 5.30^a^	12.40 ± 2.56^a^, ^b^	2.35 ± 0.900^b^, ^c^
Caspase‐3 (ng/mg protein)	1.10 ± 0.368^a^, ^b^, ^c^	3.41 ± 0.692^a^	1.92 ± 0.717^a^, ^b^	1.13 ± 0.327^b^, ^c^
TNF‐α (ng/mg protein)	13.90 ± 3.97^a^, ^b^, ^c^	100.10 ± 21.20^a^	65.10 ± 18.30^a^, ^b^	13.40 ± 5.03^b^, ^c^

*Note: p* is given according to the “one‐way ANOVA” test. Post hoc evaluations within the group were made with the “Tukey” test (*p* < 0.05).

^a^ It is significantly different from the control group.

^b^ From the DOX group.

^c^ From the DOX + ARPM (400 mg/kg) group.

It was observed that the expression of the GRP78 protein escalated approximately 16‐fold in the DOX group compared to the control group, yet notably decreased in the mixture treatment group (*p* < 0.01). There was no significant change in the protein expression in the mixture group (ARPM). Similar to our study, in ERS induced by cisplatin, GRP78 protein expression increased approximately threefold in the toxicity group compared to the control group, but in the presence of an antioxidant substance (ethyl pyruvate), it was reduced [43]. Reportedly, the GRP78 serves as a central regulator of ERS. Under conditions of cellular stress, the primary function of GRP78 protein is to facilitate the correct folding of misfolded or aggregated proteins, thereby restoring cellular homeostasis. GRP78 functions as one of the primary defense mechanisms against ERS by recognizing misfolded proteins and initiating the UPR pathways. During this process, GRP78 activates the IRE1α, PERK, and ATF6 pathways. In addition, it is a significant antiapoptotic and anti‐stress protein that prevents apoptosis and mitigates the effects of oxidative stress. Similar to our study, it was reported that the amount of GRP78 protein increased approximately fourfold in ovarian tissues induced by oxidative stress with cisplatin but decreased in the presence of antioxidant substances [43, 44]. Hence, it plays a pivotal role in regulating cellular response and becomes a critical target in various pathological conditions such as cancer, neurodegenerative diseases, and diabetes [6, 44].

The CHOP is a transcription factor that plays a crucial role in the ERS response. Under ERS conditions, imbalances in protein folding or the accumulation of stress factors within the cell trigger CHOP activation, like other GRP78 and IRE1 proteins [45, 46]. The expression of CHOP was markedly elevated in the DOX group, whereas it significantly decreased in the treatment group (*p* < 0.01). No significant change was observed in the expression of CHOP in the mixture group (Table 3). In a study investigating testicular ERS, findings consistent with our research demonstrated a significant reduction in CHOP protein levels in the gentisic acid‐treated group subjected to cisplatin‐induced testicular damage [12]. Consequently, CHOP has been identified as a key determinant in the cellular decision‐making process between survival and apoptosis under stress conditions. Excessive or prolonged activation of CHOP can lead to cellular damage and is implicated in various pathological conditions, including neurodegenerative diseases, diabetes, and cardiovascular diseases [47, 48].

Caspase‐3 activity was measured to assess apoptotic changes in the experimental groups, with the results presented in Table 3. The caspase‐3 activity increased about threefold in the DOX group and was observed to have a significant decrease in the treatment group (*p* < 0.01). The enzyme is a key mediator of cellular programmed cell death, and upon activation, caspase‐3 initiates the apoptotic pathway by proteolytically cleaving numerous substrates within the cell, ultimately leading to programmed cell death [47]. This includes the cleavage of structural proteins in the cytoskeleton and nuclear proteins, as well as the activation of other enzymes that contribute to the breakdown of the cell. Ultimately, caspase‐3 activity results in the characteristic morphological and biochemical changes associated with apoptosis, such as cell shrinkage, DNA fragmentation, and the formation of apoptotic bodies [48]. In an experimental animal study with cisplatin, a different chemotherapy drug, it was found that the caspase‐3 enzyme showed a significant increase in the toxicity group in the cisplatin group, but a significant decrease in the cisplatin group given with gentisic acid, a polyphenol rich in antioxidant value [14]. The most important common features of semi‐herbal bee products, such as honey, pollen, and propolis in terms of biological activity, are the secondary metabolites in their structures. Some flavonoids such as caffeic acid and caffeic acid phenethyl ester, quercetin, pinocembrin, which are important members of the polyphenols family, are important metabolites in terms of antioxidant, anti‐inflammatory and antitumor and are also important components of this natural mixture [49]. Quercetin is also named as a promising cancer chemopreventive agent, although it inhibits ERS in ovarian cancer cells, interestingly, it has also been shown to cause apoptosis. However, further functional studies have shown that quercetin‐induced ERS has been shown to simultaneously activate protective autophagy in studies [42]. Like our study, in a toxicity study conducted with cisplatin, it was shown that Caspase activity increased approximately eightfold in the cisplatin group, but in the experimental groups in which gallic acid (2.5 and 5 mg/kg) was administered together with cisplatin, caspase‐3 activity decreased depending on the amount of antioxidant substance [16].

In the study, TNF‐α was examined as a marker of inflammation (Table 3). While a significant increase was detected in the DOX group, it was found to decrease significantly in the treatment group (*p* < 0.01). No change was detected in the mixture group. TNF‐α protein plays a significant role in the immune system and inflammation processes. It is secreted in response to various stimuli such as cellular stress, infections, or tissue damage. TNF‐α serves as a crucial mediator in intercellular communication to regulate various biological responses, including cell death [50]. In addition, dysregulation or excessive activation of TNF‐α can play a role in many inflammatory diseases, autoimmune disorders, and conditions like cancer. TNF‐α levels are measured in biological samples (typically expressed as ng per mg of protein) to assess inflammatory processes and diseases [50]. In experimental ERS induced by cisplatin, it was reported that TNF‐α was significantly increased in the toxicity group, but inflammation decreased in the presence of antioxidant substances [14, 18]. However, no change was observed in the mixture group. In all experimental groups, it was determined that there was no change in ERS parameters, anti‐inflammatory, and apoptotic activity in Group 4, which is the ARPM administered rats.

In the study, estradiol (E2) and PGN levels as hormone biomarkers were measured using an ELISA kit. (Table 4). Significant decreases and increases in estradiol and PGN hormones were recorded in the DOX group. It was found that both hormone levels increased significantly in the group to which this mixture was applied together with DOX (*p* < 0.01). Estradiol is primarily produced in the ovaries, specifically by the granulosa cells of the ovarian follicles. PGN is also primarily produced in ovaries, particularly by the corpus luteum, a temporary endocrine structure that forms after ovulation. The reduction in both hormone levels may be a significant indicator of ovarian tissue damage caused by ERS induced by the chemotherapy drug [7].

**TABLE 4:** The serum estradiol and progesterone amounts in the rats.

Parameters	Control (saline) (*n* = 9)	DOX (6 mg/kg) (*n* = 11)	DOX + ARPM (400 mg/kg) (*n* = 11)	ARPM (400 mg/kg) (*n* = 11)
Estradiol (E2) (ng/L)	830 ± 104^a^, ^b^, ^c^	190 ± 48.0^a^	514 ± 89.07^a^, ^b^	789 ± 40.0^b^, ^c^
Progesterone (ng/mL)	9.23 ± 1.21^a^, ^b^, ^c^	3.83 ± 1.60^a^	5.29 ± 1.99^a^	7.00 ± 1.68^b^, ^c^

*Note: p* is given according to the “one‐way ANOVA” test. Post hoc evaluations within the group were made with the “Tukey” test (*p* < 0.05).

^a^ It is significantly different from the control group.

^b^ From the DOX group.

^c^ From the DOX + POHPOR (400 mg/kg) group.

In addition, no animal mortality was observed in the experimental groups treated with the formulated ARPM. This finding suggests that the mixture is non‐toxic and well‐tolerated in the administered doses, supporting its safety for further therapeutic applications. The components of the mixture‐honey, pollen, propolis, and royal jelly are known for their nutritional and nutraceutical properties, thus no toxic effects were anticipated. These natural products are widely recognized for their bioactive compounds, which contribute to their safety when used in therapeutic applications.

### 3.4 Antioxidant Parameters in Tissue

Intracellular glutathione (GSH), superoxide dismutase (SOD), and lipid peroxidation values are important markers of oxidative damage in ovarian tissues. In the study, it was determined that the level of reducing GSH was significantly decreased in the DOX group and increased in the treatment group (Table 5). GSH is an antioxidant molecule and is also an important coenzyme of enzymes such as GSH reductase and glutathione *S*‐transferase [49]. Similar to GSH concentration, SOD activity was significantly decreased in the DOX group but increased in the treatment group. In an experimental ERS study with cisplatin, it was shown that cisplatin decreased SOD activity, but activity increased in the gentisic acid treatment group [14]. SOD is an important antioxidant enzyme and eliminates oxidant stress by using GSH in the elimination of oxidant agents. Various lipid peroxides are formed because of oxidative stress, one of which is MDA [50]. In our study, it was found that MDA increased approximately 10‐fold in the DOX group but decreased in the treatment group. In a study similar to ours, it was reported that cisplatin increased MDA formation in ovaries, but gallic acid, an antioxidant component, decreased in the treatment group [14]. This ARPM, rich in polyphenols, used in the study, shows that the increased MDA level as a result of DOX‐induced oxidative cell damage is significantly reduced. Many experimental studies have reported that flavonoids such as luteolin, quercetin, and myricetin prevent cancer formation by reducing mitochondrial stress [16, 42, 54].

**TABLE 5:** The antioxidant, oxidant, and inflammation parameters in the ovarian tissues of the rats.

Parameters	Control (saline) (*n* = 7)	DOX (6 mg/kg) (*n* = 11)	DOX + ARPM (400 mg/kg) (*n* = 11)	ARPM (400 mg/kg) (*n* = 11)
GSH (ng/mg protein)	119.00 ± 8.94^a^, ^b^, ^c^	52.60 ± 25.9^a^	85.10 ± 12.4^a^, ^b^	116.00 ± 14.7^b^, ^c^
SOD (ng/mg protein)	12.20 ± 2.14^a^, ^b^, ^c^	1.92 ± 0.820^a^	5.12 ± 1.06^a^, ^b^	11.10 ± 1.57^b^, ^c^
MDA (nmol/mg protein)	4.26 ± 1.87^a^, ^b^, ^c^	34.70 ± 13.4^a^, ^b^	19.20 ± 4.33^a^, ^b^	6.73 ± 1.99^b^, ^c^

*Note: p* is given according to the “one‐way ANOVA” test. Post hoc evaluations within the group were made with the “Tukey” test (*p* < 0.05).

^a^ It is significantly different from the control group.

^b^ From the DOX group.

^c^ From the DOX + POHPOR (400 mg/kg) group.

In studies involving the chrysin flavonoid, an important component of this mixture, it has been demonstrated that chrysin protects ovarian tissues from damage in rats subjected to experimental oxidative stress. Chrysin is believed to exert its effects by reducing oxidative stress and activating the estrogen receptor‐beta (ERβ) pathway [55, 56]. Similarly, pinocembrin has been reported to protect against ovarian cancer by downregulating the mRNA levels of N‐cadherin and GABAB receptors [57]. Clinical studies have reported that caffeic acid and CAPE, key components of propolis, are effective in modulating ERS pathways and in safeguarding reproductive tissues from damage [58].

### 3.5 Histological Evaluations

**FIGURE 1:**
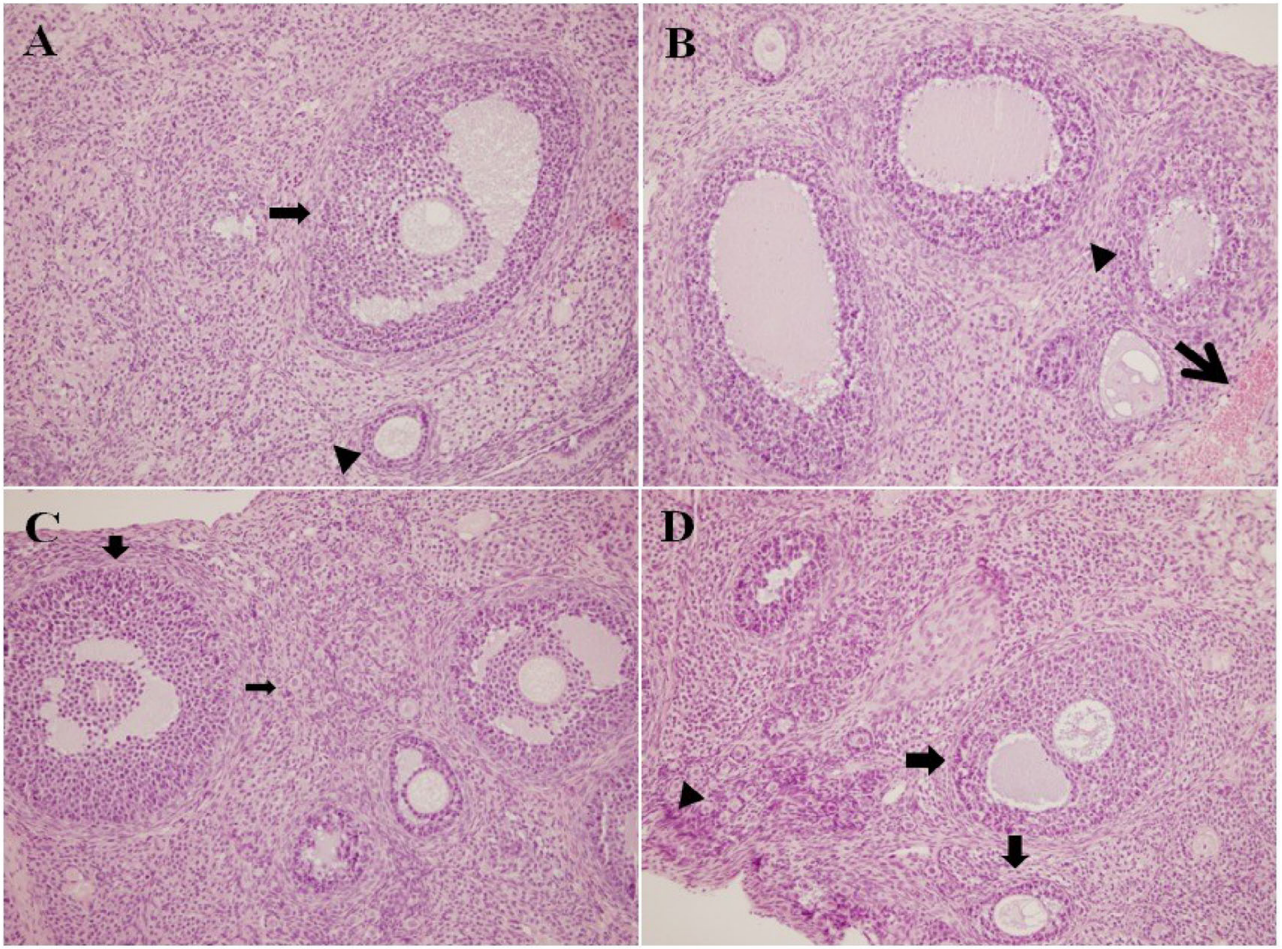
Morphological examination of rat ovarian tissues (H&E, ×200). (A) Control group: Ovarian tissues from the control group exhibited normal ovarian secondary, and tertiary follicles were widely observed in the cortex. (B) DOX group: Ovarian tissue from the DOX group exhibited severe vascular congestion and widespread degenerative changes, an atretic follicle structures. (C) DOX + ARPM group: Ovarian tissues exhibited primary, secondary and tertiary follicles, and small atretic changes. (D) ARPM group: Ovarian tissues exhibited slight irregularities in the germinal epithelium (arrow) but were close to a normal morphology. Intermittent mild atretic changes were observed in ovarian follicles (arrowhead). Primordial, primary, secondary, and tertiary follicles were close to normal numbers and morphology in the tissue.

**FIGURE 2:**
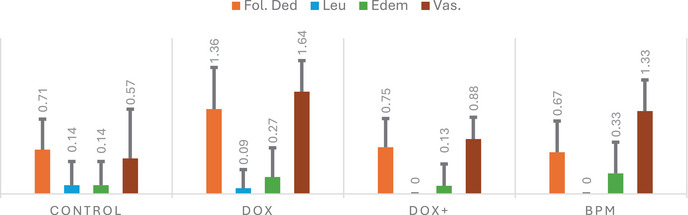
Histopathological evaluation of the ovarian tissue's follicular degeneration (Fol. deg), leukocyte (lec.), edema (Ede), vascular congestion (Vas.). *p* values according to Kruskal–Wallis's variance analysis (the Mann–Whitney *U* test post hoc). Data expressed as median (interquartile range for 25%–75%) values.

To assess pathological changes in tissues and cellular damage, parameters including follicular cell degeneration, leukocyte infiltration, edema, and vascular congestion were examined. The histopathological features of the ovarian tissue sections from the four study groups are presented in Figure 1. Treatment with the mixture (400 mg/kg) significantly improved follicle cell degeneration (*p* < 0.001), leukocyte (*p* < 0.02), edema (*p* < 0.014), and vascular congestion (0.001) in ovarian cells compared to the DOX group (*p* < 0.001) (Figure 2). Follicular cell degeneration, which is used as an indicator of cellular damage and apoptotic processes, means the deterioration of the structural integrity and functions of the cells [59]. Follicular degeneration was found to be significantly increased in the DOX group, whereas it was notably reduced in the treatment group. The presence of leukocytes in the tissues is an important parameter indicating the degree of inflammatory response, and it was determined that the presence of leukocytes increased in the DOX group but decreased in the treatment group (DOX). Edema is a tissue damage that refers to the accumulation of fluid between cells and usually occurs as a result of inflammation, trauma or infection, and is considered an indicator of cellular dysfunction

It was determined that edema increased in the DOX group and decreased in the group treated with the mixture compared to the control group. Vascular congestion is defined as abnormal blood accumulation in blood vessels and may develop due to inflammation or toxic effects. In short, this parameter helps to evaluate whether blood flow in the tissue is affected and the resulting hypoxia. Vascular congestion was found to increase significantly in the DOX group but decrease in the mixture group.

**FIGURE 3:**
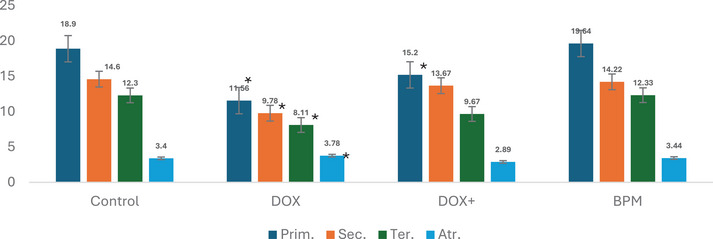
Evaluation of the ovarian reserves. Data was expressed as mean ± SD. Inter‐group relationships were assessed using one‐way ANOVA, followed by Tukey's and Tamhane's post hoc tests. Statistical significance was set from the control group *< 0.05. Atr., atretic follicles; Prim., primary follicles; Sec., secondary follicles; Ter., tertiary follicles.

Primary, secondary, tertiary, and atretic follicles are parameters that express different stages of ovarian follicle development during oogenesis, indicating the process by which oocytes (eggs) are produced in the ovaries [29]. Figure 3 shows the oocyte cell counts of the experimental groups. In the DOX group, a decrease in the number of primary, secondary, and tertiary follicles, along with an increase in the number of atretic follicles, was observed compared to the control group. However, due to large standard deviation values, these changes were determined to be statistically insignificant. Although an increase in the number of ovulations and a decrease in atretic follicles were observed in the groups receiving only this mixture, these changes were not found to be statistically significant.

This study demonstrated that the chemotherapy drug DOX induces significant oxidative stress, leading to severe ERS and subsequent damage to ovarian tissues. It was found that an ARPM with high antioxidant, anti‐inflammatory, and cytotoxic activities played a crucial role in mitigating this stress and significantly contributed to the recovery of ovarian tissues.

## 4 Conclusions

In this study, the protective potential of an ARPM containing oak honey, chestnut pollen, propolis, and royal jelly against oxidative stress was evaluated in vitro an in vivo. The mixture was found to be highly effective in preventing ovarian damage caused by oxidative stress induced by DOX, a chemotherapy agent, in experimental animals. ARPM mixture was found to have an effect on ovarian tissue through the ERS pathway and by protecting the hormonal system. In conclusion, ARPM formulation, a functional food supplement, may have an important supplementary role in protecting reproductive health against chemotherapy‐induced ovarian damage. Further research is warranted to explore its broader applications and to elucidate the mechanisms underlying its protective effects.

## Author Contributions

**Meltem Arıkan Malkoç**: obtaining animal ethics committee permission, animal trials, biochemical analyses. **Serap Özer Yaman**: experimental animal studies, hormone analyses, statistical studies. **Şafak Ersöz**: histopathology studies. **Sevgi Kolaylı**: planning the study.

## Conflicts of Interest

The authors declare no conflicts of interest.

## Data Availability

Data will be made available on request.
